# Gene-knockdown Methods for Silencing Nuclear-localized Insulin Receptors in Lung Adenocarcinoma Cells: A Bioinformatics Approach

**DOI:** 10.2174/0113892029298721240627095839

**Published:** 2024-07-03

**Authors:** Qiu Ren, Hui Ma, Lingling Wang, Jiayu Qin, Miao Tian, Wei Zhang

**Affiliations:** 1 Department of Respiratory and Critical Care Medicine, The First Affiliated Hospital of Harbin Medical University, No.23 Post Street, Nangang District, Harbin 150001, China;; 2 Department of Respiratory, Heilongjiang Province Hospital Harbin, Harbin, 150000, China;; 3 Department of Respiratory Medicine, First Affiliated Hospital of Harbin Medical University, Harbin, 150001, China;; 4 Heilongjiang College of Business and Technology. Harbin, Heilongjiang, People's Republic of China

**Keywords:** Lung adenocarcinoma, ERK signaling pathway, IL-1B, bioinformatics, genes, immunoblotting assays

## Abstract

**Background:**

Lung adenocarcinoma, the predominant subtype of lung cancer, presents a significant challenge to public health due to its notably low five-year survival rate. Recent epidemiological data highlights a concerning trend: patients with pulmonary adenocarcinoma and comorbid diabetes exhibit substantially elevated mortality rates compared to those without diabetes, suggesting a potential link between hyperinsulinemia in diabetic individuals and accelerated progression of pulmonary adenocarcinoma. Insulin Receptor (IR) is a tyrosine-protein kinase on the cell surface, and its over-expression is considered the pathological hallmark of hyperinsulinemia in various cancer cell types. Research indicates that IR can translocate to the nucleus of lung adenocarcinoma cells to promote their proliferation, but its precise molecular targets remain unclear. This study aims to silence IRs in lung adenocarcinoma cells and identify key genes within the ERK pathway that may serve as potential molecular targets for intervention.

**Methods:**

Gene expression data from lung adenocarcinoma and para cancer tissues were retrieved from the Gene Expression Omnibus (GEO) database and assessed through "pheatmap", GO annotation, KEGG analysis, R calculations, Cytoscape mapping, and Hub gene screening. Significant genes were visualized using the ggplot2 tool to compare expression patterns between the two groups. Additionally, survival analysis was performed using the R "survminer" and "survival" packages, along with the R "pathview" package for pathway visualization. Marker genes were identified and linked to relevant signaling pathways. Validation was conducted utilizing real-time quantitative polymerase chain reaction and immunoblotting assays in an A549 lung cancer cell model to determine the roles of these marker genes in associated signaling cascades.

**Results:**

The study examined 58 lung adenocarcinoma samples and paired para-neoplastic tissues. Analysis of the GSE32863 dataset from GEO revealed 1040 differentially expressed genes, with 421 up-regulated and 619 down-regulated. Visualization of these differences identified 172 significant alterations, comprising 141 up-regulated and 31 down-regulated genes. Functional enrichment analysis using Gene Ontology (GO) revealed 56 molecular functions, 77 cellular components, and 816 biological processes. KEGG analysis identified 17 strongly enriched functions, including cytokine interactions and tumor necrosis factor signaling. Moreover, the ERK signaling pathway was associated with four Hub genes (FGFR4, ANGPT1, TEK, and IL1B) in cellular biological processes. Further validation demonstrated a positive correlation between IL-1B expression in the ERK signaling pathway and lung cancer through real-time fluorescence quantitative enzyme-linked reaction with immunoblotting assays.

**Conclusion:**

In IR-silenced lung adenocarcinoma, the expression of the IL-1B gene exhibited a positive correlation with the ERK signaling pathway.

## 1 INTRODUCTION

Lung cancer is one of the most lethal diseases globally. GLOBOCAN, the International Agency for Cancer Research, has estimated that there will be about 13 million new cancer cases by 2040. Currently, lung cancer ranks as the most prevalent cancer, constituting 11.6% of all cancer cases and the leading cause of cancer-related mortality, representing 18.4% of all cancer cases [1]. Histologically, lung cancer is classified into two primary types: non-small cell lung cancer (NSCLC; approximately 85% of all lung cancer cases) and small cell lung cancer [2]. The lack of suitable diagnostic markers for early detection significantly impedes diagnostic rate and NSCLC treatment efficacy. Consequently, most patients receive their diagnosis at an advanced stage, with half already presenting with distant metastatic disease upon initial diagnosis [3]. Over the past decade, significant developments have been made in chemotherapy, radiotherapy, surgery, and targeted therapy for lung cancer. In particular, there have been notable advancements in molecularly targeted therapy for NSCLC. Recent breakthroughs in molecular biology have also contributed to enhancing the precision of diagnosis and treatment, thereby driving further research in NSCLC.

Inflammation serves as the body's innate response to infection and injury. However, in chronic conditions such as cancer, persistent inflammation paradoxically promotes tumor growth, and pro-tumor inflammation is now acknowledged as a hallmark of cancer because it enables neoplastic tumors to acquire additional disease characteristics [4]. Various inflammatory pathways, including the IL-1B and TNF-α pathways, have been implicated in tumorigenesis [5, 6]. Inflammatory vesicles, comprising a multiprotein complex, trigger downstream inflammatory pathways in response to endogenous danger signals, thereby activating IL-1B. Substantial preclinical evidence supports the involvement of IL-1B in cancer progression and development, such as tumor initiation, promotion, angiogenesis, and metastasis [7, 8]. Wong *et al.* demonstrated a significant reduction in lung cancer incidence and mortality by employing the IL-1B inhibitor canasumab to target inflammatory cells and inhibit the IL-1B inflammatory pathway [9]. Xie *et al.* analyzed serum and exhaled breath condensate from lung cancer patients to determine IL-1B concentration and content. They reported stage-dependent variation in NSCLC concentration, positively correlating with severity and stage. Thus, IL-1B was proposed as a potential biomarker for NSCLC [10].

The Insulin Growth Factor (IGF) axis, which comprises insulin growth factors and Insulin Receptors (IRs), plays an essential role in tumor progression [11]. Activation of IR has been found to activate the PI3K/Akt and MEK/ERK signaling pathways [12], which then promote immunosuppressive and anti-inflammatory responses that support tumor growth. Additionally, activation of the MEK/ERK pathway, another downstream signaling pathway of IGF1R, correlates with tumor infiltration [13]. Collectively, these observations suggest that the activation of IR signaling may also influence the proliferation of lung cancer.

A previous study reported that nuclear-localized IR is involved in the biological function of lung cancer cells, with its action pathway associated with the ERK1/2 signaling pathway [14]. However, it did not investigate the target genes involved in this action-related signaling pathway. Systems biology can be used to predict emergent properties and behaviors of biological systems through computational models and simulations, thus connecting molecular components across various biological scales [15]. Moreover, it helps in generating new hypotheses by uncovering hidden relationships and patterns in large datasets. Therefore, this study aims to use bioinformatics methods to screen for target genes related to the ERK1/2 signaling pathway in lung cancer cells and validate the involvement of these target genes in the activity of nuclear-localized IR.

## 2 MATERIALS AND METHODS

### 2.1 Data Sources

The dataset used in this study was obtained from the open GEO GSE32863 dataset of the Lung Cancer Database, which includes protein-coding gene expression data for 58 pairs of lung adenocarcinomas and their corresponding para-neoplastic tissues. Differential expression analysis was performed using the "limma" package in R (version=3.6.1), and volcano mapping was conducted using the "ggplot2" package. Additionally, heat mapping was performed using the "pheatmap" package.

### 2.2 Functional Enrichment Analysis

Gene Ontology (GO, http://www.geneontology.org/) is a widely used bioinformatics tool for the enrichment analysis of gene sets, including Biological Processes (BP), Cellular Components (CC), and Molecular Functions (MF). The Kyoto Encyclopedia of Genes and Genomes (KEGG, http://www.genome.jp/kegg/) is a database for investigating enrichment pathways of selected genes aimed at enhancing our comprehension of gene function. In this study, a Q value < 0.05 and gene counts ≥ 10 were considered statistically significant.

### 2.3 Signaling Pathway Gene Screening

The R software (version=3.6.1) was used to identify ERK-related pathways and extract related genes. A False Discovery Rate (FDR) threshold of 0.01 and a threshold of |logFC| ≥ 1 were applied. Then, differentially expressed genes were screened, and volcano plots and heatmaps were generated. Co-expression regulatory networks were mapped using Cytoscape software, and hub genes were identified. A significance threshold of *p* < 0.05 and a correlation coefficient (cor) > 0.6 were considered statistically significant.

### 2.4 Analysis of Expression Differences Between Key Genomes

In the analysis, the R(version=3.6.1) language "ggplot2" package was utilized to visualize the expression patterns of 11 hub genes across key terms between the two groups. A half-violin plot was generated to depict the distribution of gene expression levels, providing insights into the variation and trends within each group.

### 2.5 TCGA-LUAD Gene Expression Data

Gene expression data from 58 para-neoplastic and 510 cancer tissue samples from the TCGA-LUAD database were analyzed. Eleven hub genes were subjected to standard t-tests, and heat maps were plotted to visualize the expression patterns across the samples.

### 2.6 Survival Analysis

Survival analysis was conducted using the "survivor" and "survminer" packages in R, and survival curves of the high- and low-risk groups were plotted accordingly.

### 2.7 Cell Culture

The human NSCLC A549 cell line was purchased from the American Type Culture Collection (ATCC) and cultured in DMEM medium supplemented with 10% FBS, 100 U/ml penicillin, 100 I1/4g/ml streptomycin and 2 mM L-glutamine. Cell line origin: Procell Life Science & Technology Co, Ltd. Wuhan, China. Mycoplasma detection: The cell line company has already conducted STR identification in 2022 when the cell line was purchased.

### 2.8 Cell Transfection

A549 cells were digested and attached to culture plates at a density of 1x10^5^ cells/ml and then placed in a cell incubator. A549 cells were transfected with 30 nM siRNAs targeting the nuclear-localized Insulin Receptor (INSR) and an siRNA(5,GAG GCT GCA CTG TGA TCA A3,NC:5,GAG GCU GCA CUG UGA UCA3,) control using Lipofectamine 3000 following the manufacturer's instructions.

### 2.9 Western Blot Experiments (WB)

Total protein from the cell samples was extracted using the RIPA lysis buffer (100 I1/4l RIPA + 1 I1/4l PMSF), and the protein concentration was determined using the BCA protein assay kit. Then, the protein samples were subjected to SDS-PAGE (4-12%) and transferred to low-fluorescence PVDF membranes. After washing the membranes three times, the membranes were fast-sealed with Fast Blocking Solution for 20 minutes. Next, the low-fluorescence PVDF membranes were incubated with primary antibodies overnight at 4°C. After three washes in TBS-0.1% Tween-20, the corresponding secondary antibody was added and incubated for 1 hour at room temperature on a slow shaker. Lastly, the membranes were rinsed three times with TBST and detected using a fluorescent imaging system (Bio-Rad).

### 2.10 Real-time Quantitative Polymerase Chain Reaction (RT-PCR)

The transfected A549 cells with INSR-siRNA were isolated from lung cancer cells using a TRIzol chloroform-based method. After washing in PBS, adherent lung cancer cells were lysed with 1 mL TRIzol, and chloroform (200 I1/4L) was added for phase separation. After centrifugation, the aqueous phase containing RNA was precipitated with isopropanol, and RNA pellets were washed with 75% ethanol. RNA concentration was determined by A260 absorbance. Reverse transcription of 2 I1/4g total RNA was performed to synthesize the first cDNA strand. Quantitative real-time RT-PCR using SYBR Green included GAPDH as an internal control. The primer sequences used were as follows: GAPDH (Forward: ACTCCCATTCTTCCACCTTTG, Reverse: CCCTGTTGCTGTAGCCATATT), IL-1B mRNA (Forward: GGCCCTAAACAGATGAAGTGCT, Reverse: TGTCCATGGCCACAACAACT), ERK1 (Forward: GTCAGACTCCAAAGCCCTTGAC, Reverse: AGCCGCTCCTTAGGTAGGTC), ERK2 (Forward: TACGGCATGGTGTGCTCTG, Reverse: TTGCTCGATGGTTGGTGCTC), and INSR (Forward: GGCGATATGGTGATGAGGAGC, Reverse: GTCTGTCACGTAGAAATAGGTGGG).

### 2.11 Statistical Analysis

All statistical analyses were conducted using the R software package. Two-tailed, unpaired T-tests were used to compare two groups, and t-test and one-way analysis of variance (ANOVA) followed by Tukey's multiple-comparison tests, and FDR was used for calibration were used to more than two groups, with statistical significance set at p ≤ 0.05.

## 3 RESULTS

**Fig. (1):**
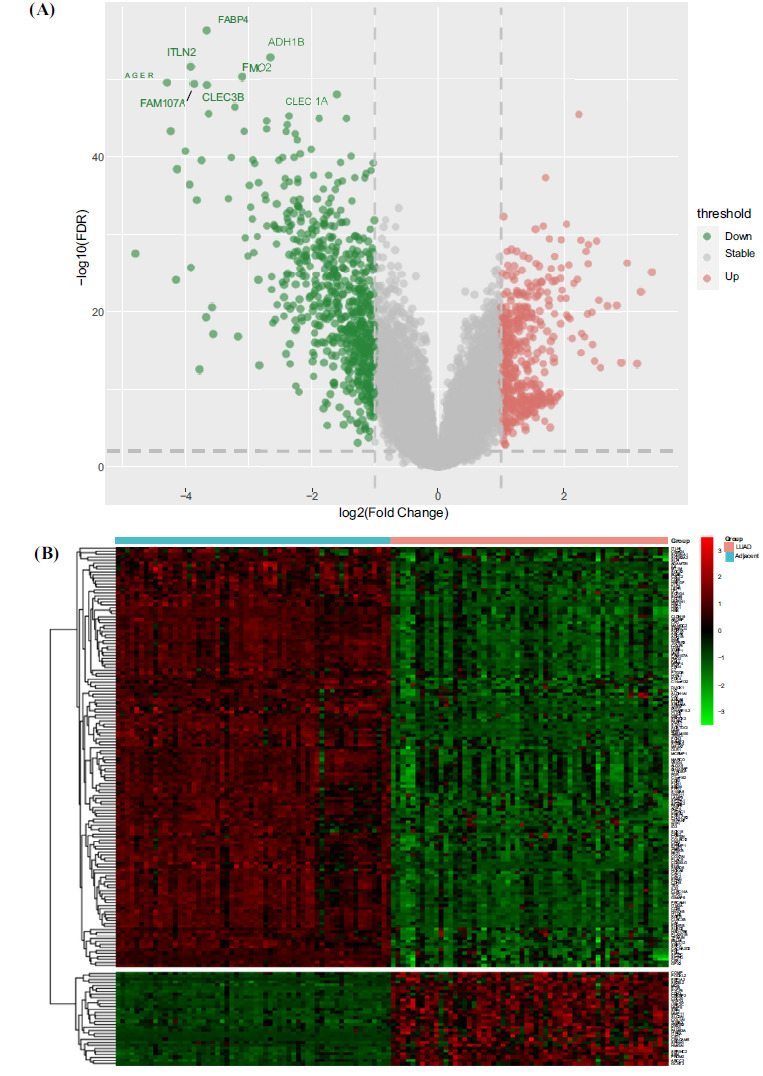
Protein-coding gene expression data analysis in lung adenocarcinoma and its paired paracancerous tissues. (**A**) Volcano plot displaying differentially expressed genes. The horizontal axis represents log2 transformed fold change values, while the vertical axis represents negative log10 transformed false discovery rate (FDR) values. Red indicates up-regulated genes, green indicates down-regulated genes, and gray indicates genes with insignificant differences. (**B**) Heat map depicting significantly different gene expression. The vertical axis represents gene names, and the horizontal axis represents lung adenocarcinoma and control samples. Red denotes high expression values, while green indicates low expression values. The upper section illustrates down-regulated genes in lung adenocarcinoma, whereas the lower section represents up-regulated genes in lung adenocarcinoma.

We utilized the GSE32863 dataset from the GEO database using the following parameters: an FDR threshold of 0.01 and a |logFC| threshold of 1. A total of 1040 genes were examined for differential expression. Then, a volcano plot was generated, revealing 421 up-regulated genes (logFC > 1) and 619 down-regulated genes (logFC < -1) (Fig. **1A**). Furthermore, the top 172 significantly differentially expressed genes were selected for visualization, employing parameters of FDR < 0.01 and |logFC| > 2, comprising 141 up-regulated genes and 31 down-regulated genes. A heatmap was then plotted accordingly (Fig. **1B**).

**Fig. (2):**
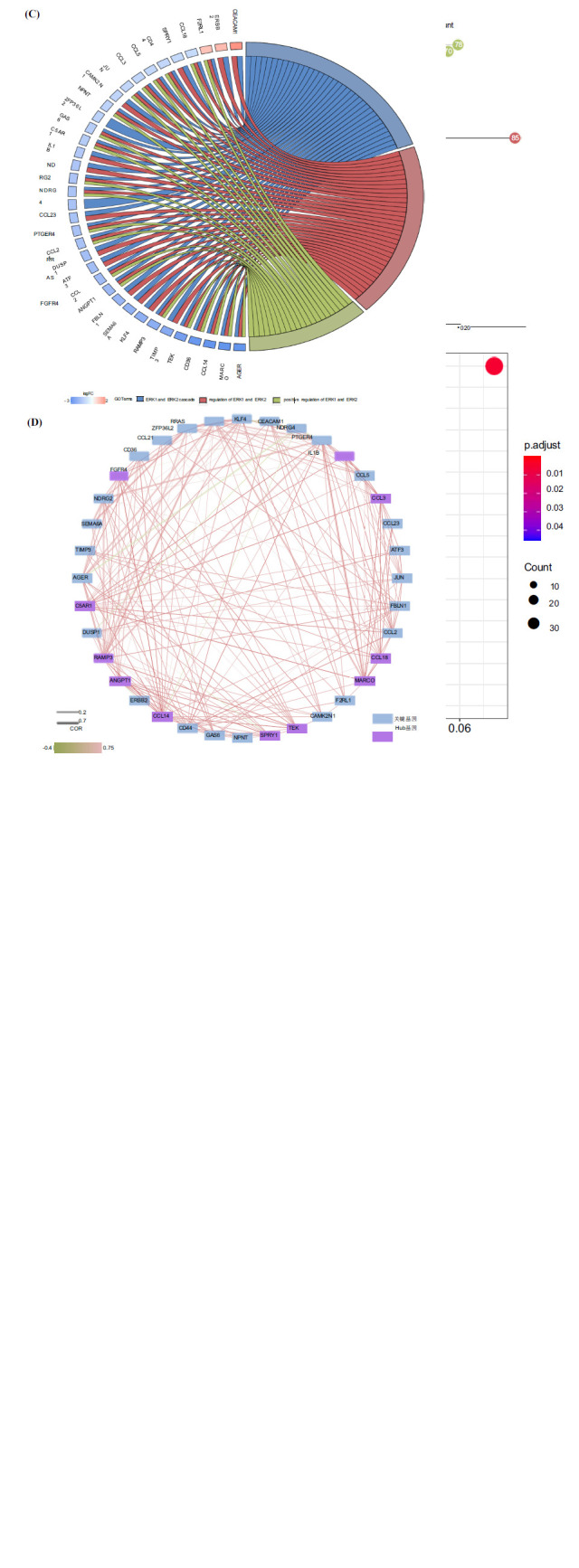
Analysis of differentially expressed genes through Gene Ontology (GO) annotation and Kyoto Encyclopedia of Genes and Genomes (KEGG) functional enrichment analysis, focusing on key biological processes. (**A**) Lollipop plot illustrating functional enrichment analysis. The vertical axis denotes enriched GO terms, while the horizontal axis represents the negative logarithm of the false discovery rate (FDR) value. Green, red, and blue colors indicate biological processes, cellular components, and molecular functions, respectively. Numbers indicate the number of differential genes within each term. (**B**) Bubble diagram depicting KEGG enrichment analysis. The vertical axis displays KEGG pathway names, and the horizontal axis shows the proportion of genes within each pathway. The color gradient indicates the significance of the pathway, with red representing higher significance. Node size reflects the number of enriched genes within each pathway. (**C**) Illustration of key terms and gene regulation string. The right side shows three key terms, color-coded to differentiate between them. The left side presents 36 key genes, with blue and red indicating down-regulation and up-regulation, respectively. (**D**) Diagram of the co-expression regulatory network of key genes, with blue nodes representing key genes and purple nodes representing hub genes. Green and red lines signify negative and positive correlations, respectively, with thicker lines indicating stronger interactions. (**E**) Half-violin plot illustrating the expression of hub genes, with green representing the control group and red representing the lung adenocarcinoma group. Error lines and dots indicate the variability and mean values of the two groups, respectively.

GO annotation and KEGG functional enrichment analyses were conducted on the differentially expressed genes, utilizing a q value of 0.05, and the results revealed a total of 816 Biological Processes (BP), 77 Cellular Components (CC), and 56 Molecular Functions (MF) that were significantly enriched. The first 30 significant terms in each category are shown in Fig. (**2A**). Additionally, KEGG functional enrichment analysis revealed 17 significantly enriched pathways, primarily involving cytokine and cytokine receptor interactions, phagosome, and the tumor necrosis factor signaling pathway, among others (Fig. **2B**). After functional enrichment, the top three genes involved in key Biological Processes (BP) and related to the ERK signaling pathway were screened, yielding a total of 36 key genes (Fig. **2C**). The co- expression coefficients of these 36 key genes in disease samples were calculated using R, and a co-expression regulatory network was constructed using the Cytoscape software, resulting in the identification of 11 Hub genes among the 36 key genes (*p* < 0.05, cor > 0.6) (Fig. **2D**). The expression of the 11 Hub genes in key terms between the two groups was generated using the ggplot2 package and is presented in the half-violin plots shown in Fig. (**2E**).

**Fig. (3):**
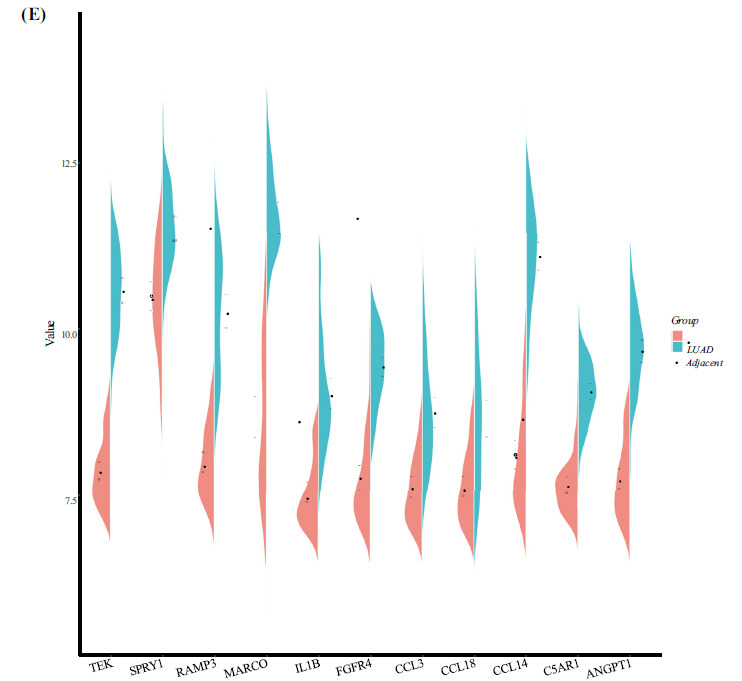
Mapping of differentially expressed genes to the MAPK signaling pathway using the path view package. Four hub genes (FGFR4, ANGPT1, TEK, IL1B) are significantly associated with ERK. The MAPK signaling pathway map depicts normalized fold change values, with colors indicating the extent of expression changes.

Given the significance of the ERK1/2 signaling pathway in regulating apoptosis, differentiation, and the cell cycle within the MAPK cascade pathways, the path view package was used to map the differential genes onto the MAPK signaling pathway, based on which four Hub genes, namely FGFR4, ANGPT1, TEK and IL1B, were identified as particularly relevant to ERK signaling (Fig. **3**).

**Fig. (4):**
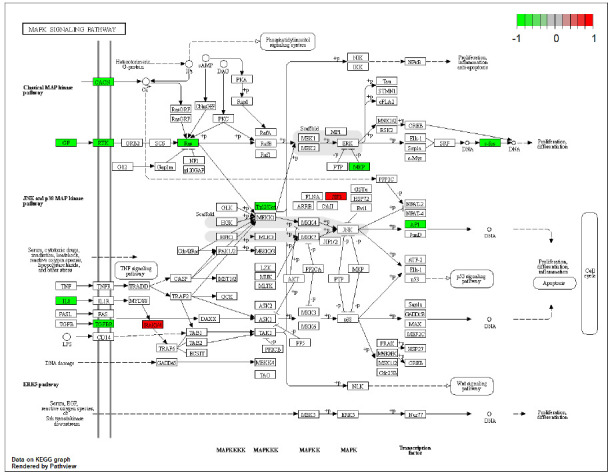
Analysis of gene expression data from the TCGA database, comprising 58 paraneoplastic and 510 cancer tissue samples. The heatmap illustrates the expression of hub genes in TCGA samples, with the vertical axis representing hub gene names and the horizontal axis indicating control and lung adenocarcinoma group samples. Red indicates high gene expression, while green signifies low gene expression.

Gene expression data from the TCGA database comprising 58 para-neoplastic and 510 cancer tissue samples were used to validate the differential expression of the 11 Hub key genes. Standard t-tests were applied to the expression levels of the 11 key genes, revealing that 10 of them exhibited significant differential expression (*p* < 0.01, |logFC| > 0) (Fig. **4**).

**Fig. (5):**
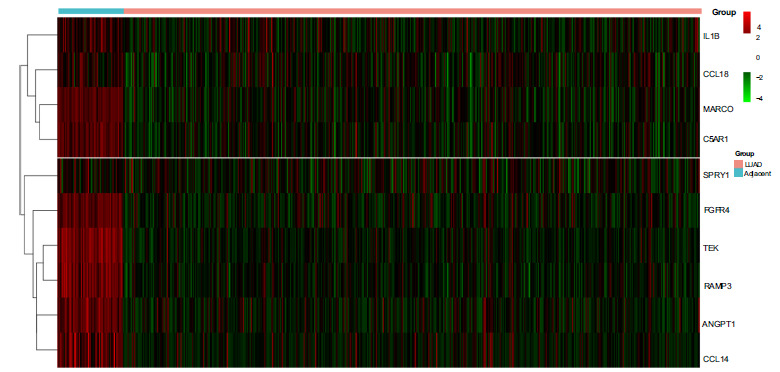
Validation of the relationship between pivotal genes and overall survival using TCGA data. Kaplan-Meier plot depicting survival analysis of marker genes. The high expression group is represented in red, the low expression group in green, and shading indicates the 95% confidence interval. Strata represents the number of disease samples included within the two groups.

In the TCGA-LUAD dataset, gene expression data were utilized to determine the correlation between hub genes and overall survival. The "Survminer" and "survival" packages were used to analyze gene expression levels and survival outcomes. Survival analysis revealed that 6 hub genes, defined as marker genes, exhibited significant associations with survival (*p* < 0.05) (Fig. **5**).

**Fig. (6):**
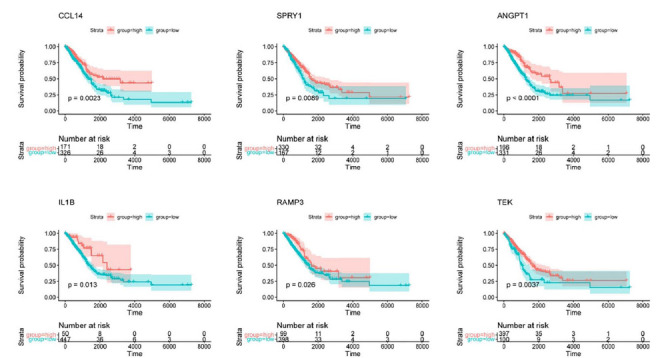
IL-1B protein expression and mRNA expression in the ERK1/2 signaling pathway after silencing INSR in A549 cells. (**A**) ERK agonist (LM22B-10) promotes IL-1B expression in siINSR-treated cells. (**B**) Comparison with the control group reveals statistically significant differences in IL-1B expression (*P<0.05, **P<0.01). (**C-F**) mRNA levels of ERK1, ERK2, IL-1B, and INSR in siINSR-treated cells. Statistically significant differences are denoted as *P<0.05, **P<0.01, ***P<0.001.

Furthermore, the silencing of INSR in A549 cells resulted in elevated IL-1B expression within the ERK1/2 signaling pathway. The immunoblotting experiments were categorized into three groups: the first group comprised A549 cells treated with siRNA control (NC), the second group comprised A549 cells treated with INSR siRNA, and the third group comprised A549 cells treated with INSR siRNA and subsequently exposed to ERK agonist (LM22B-10). The results indicated that IL-1B expression in the ERK1/2 signaling pathway was significantly reduced in the second group compared to the first group. Following the addition of the ERK agonist (LM22B-10), IL-1B expression increased compared to the second group. These results indicate a potential modulation by IL-1B on the biological activity of lung cancer cells with nuclear-localized IRs, potentially through the ERK signaling pathway (Figs. **6A** and **B**).

Additionally, mRNA expression levels were verified through polymerase chain reaction experiments in three experimental groups (group I: A549 cells+siRNA+NC group; II: A549cells +siRNA+INSR; and III: A549cells+siRNA-INSR + LM22B-10), and the results showed that group II had reduced expression levels of ERK1, ERK2, and IL-1B mRNA compared to group III, which was treated with INSR siRNA and subsequently exposed to the ERK agonist (LM22B-10). Moreover, the expression of INSR mRNA was reduced in experimental group II compared to experimental group I. Notably, both INSR mRNA expression and IL-1B mRNA expression levels were decreased in experimental group II and experimental group III compared to experimental group I (*p* < 0.05, *p* < 0.01) (Figs. **6C-F**).

## 4 DISCUSSION

Lung cancer is one of the most prevalent malignant tumors worldwide, characterized by a notably high mortality rate. Hence, investigating factors to target these cancer cells holds paramount importance in improving patient treatment outcomes. Previous studies have suggested that the nuclear-localized IR contributes to the proliferation and migration of lung cancer cells, likely *via* the ERK1/2 signaling pathway. Therefore, elucidating its involvement in signaling pathway-related target genes could have important clinical significance.

The Mitogen-Activated Protein Kinase (MAPK) cascade is an important signaling pathway through which many important cellular functions occur, including cell proliferation, metabolism, differentiation, DNA repair, and apoptosis [16, 17]. Among the various MAPK cascade pathways, the ERK1/2 signaling pathway is the most extensively studied and has been intricately involved in apoptosis, differentiation, and cell cycle regulation [18]. Dysregulation or mutations in ERK1/2 signaling factors have been associated with drug resistance and various cancers, including lung cancer [19]. ERK functions as a serine/threonine protein kinase and is a key player in signal transduction, particularly in transmitting mitogenic signals [20]. Typically located in the cytoplasm, ERK translocates to the nucleus upon activation, where it modulates transcription factor activity and gene expression [21]. While ERK1/2 predominantly resides in the cytoplasm of unstimulated cells, their nuclear translocation post-activation regulates various transcription factors *via* phosphorylation, consequently modulating cellular metabolism and functions and exerting specific biological effects [22]. Notably, ERK has been reported to be up-regulated in various human tumors, including ovarian, colon, breast, lung, and hematologic malignancies [23]. Active ERK is involved in numerous cellular processes such as apoptosis, DNA repair, cell cycle regulation, autophagy, and senescence. The ERK/MAPK pathway is the primary signaling cascade regulating cell growth and proliferation, and due to its near-ubiquitous activation in cancers, mutations that activate this pathway are the most prevalent oncogenic factor across different malignancies [24].

IL1B, a proinflammatory cytokine, plays essential roles in both acute and chronic inflammation. It is secreted by lung epithelial cells and facilitates cell proliferation, differentiation, and apoptosis by up-regulating several inflammation-related genes, such as tumor necrosis factor and reactive oxygen species [25]. The IL1B gene encodes an inflammatory mediator crucial in chronic inflammation and carcinogenesis [26]. *In vitro* studies have demonstrated IL1's significance in promoting proliferation, which contributes to malignant transformation [27]. Additionally, Fibroblast Growth Factor Receptor 4 (FGFR4), a tyrosine kinase receptor for FGFs, regulates various cellular processes, including cell proliferation and differentiation.

Previous research has established that the nuclear localization of the IRs promotes the proliferation and migration of lung cancer cells, with its effects mediated *via* the ERK signaling pathway [14]. Furthermore, the target gene implicated in lung adenocarcinoma and the ERK signaling pathway was identified as IL-1B, showing a more pronounced survival curve. Subsequent *in vitro* experimental studies using A549 cells subjected to Insulin Receptor (INRS) gene silencing revealed reduced IL-1B expression compared to the control group, indicating its association with the ERK signaling pathway. Moreover, additional silencing of ERK1/2, following IR silencing, further decreased IL-1B expression compared to the control group and significantly attenuated its expression compared to non-silenced ERK1/2. Real-time quantitative PCR analysis at the mRNA level post-IR silencing also showed a lower amplification number for IL-1B mRNA. These findings confirm the association of IL-1B, a target gene of IR-silencing in lung cancer, with the ERK signaling pathway.

Our study uncovered a significant positive correlation between IL-1B expression and the ERK signaling pathway, suggesting that inhibiting the IR could potentially reduce the proliferation of lung adenocarcinoma cells. However, to establish the robustness of our results, further *in vivo* studies are essential. Moreover, employing biological methodologies, we identified several other hub genes, such as FGFR4, ANGPT1, and TEK, among others, which play essential roles in the ERK signaling pathway. These genes present promising avenues for future investigation and could provide valuable insights into the underlying mechanisms of lung adenocarcinoma progression.

## CONCLUSION

In conclusion, this is the first study to identify IL-1B as a target gene in lung cancer cells, elucidating its association with the ERK signaling pathway. These findings not only shed light on the intricate molecular mechanisms underlying lung cancer progression but also pave the way for further exploration into the broader biological functions influenced by insulin in lung cancer cells. Moreover, our study lays a solid foundation for future research aimed at developing targeted therapeutic interventions for the treatment of lung cancer.

## AUTHORS' CONTRIBUTIONS

Q R conducted the experiments, interpreted the data, prepared the figures, and wrote the manuscript. H M and JY Q contributed to the experiments and data interpretation. M T and Ling Ling Wang conducted data collation and analysis. W Z revised the manuscript. All authors contributed to the drafting of the article and provided final approval of the submitted version.

## Data Availability

The authors confirm that the data supporting the findings of this research are available within the article.

## LIST OF ABBREVIATIONS

ANOVA: Analysis of Variance
ATCC: American Type Culture Collection
BP: Biological Processes
CC: Cellular Components
FDR: False Discovery Rate
FGFR4: Fibroblast Growth Factor Receptor 4
GEO: Gene Expression Omnibus
GO: Gene Ontology
IGF: Insulin Growth Factor
INRS: Insulin Receptor
IR: Insulin Receptor
KEGG: Kyoto Encyclopedia of Genes and Genomes
MAPK: Mitogen-Activated Protein Kinase
MF: Molecular Functions
